# Review of Biomedical Image Processing

**DOI:** 10.1186/1475-925X-10-101

**Published:** 2011-11-18

**Authors:** Edward J Ciaccio

**Affiliations:** 1 Department of Medicine, Columbia University, New York, USA

## Abstract

This article is a review of the book: 'Biomedical Image Processing', by Thomas M. Deserno, which is published by Springer-Verlag. Salient information that will be useful to decide whether the book is relevant to topics of interest to the reader, and whether it might be suitable as a course textbook, are presented in the review. This includes information about the book details, a summary, the suitability of the text in course and research work, the framework of the book, its specific content, and conclusions.

## Summary

Biomedical Image Processing, by Thomas M. Deserno [1], is a text that is suitable for clinicians, scientists, and engineers interested in the important topic of how clinical and biomedical images can best be processed for features quantification, and to enhance important visual detail. It is compiled from the notes of multiple authors, all eminent scientists in their field of research. The book is 'all-in-one' in the sense that it can be used as the sole textbook for coursework on this topic, consisting of chapters devoted to image formation and enhancement, feature extraction and selection, image segmentation and classification of features, data visualization, image management and integration, and evaluation and customizing of processing methods.

The book begins with an introductory chapter by the editor which includes foundational material, followed by 22 chapters written by experts in biomedical image processing. Each chapter begins with an introductory paragraph, and includes an overview of developments in the particular area of research. Case examples are provided to reinforce the concepts.

One problem with books having multiply authored chapters is that they often lack cohesion. However, in this text, many of the chapters build upon information from prior chapters. The flow from one chapter to the next is therefore quite satisfactory. This was enabled in part by the editor providing his written introductory chapter to each of the authors as a guide, as he explains in the Preface. The authors then followed with a broadcast to the group of their proposed chapter outline and contents. This enabled cohesion in form, style, and content between the writings of each author.

The chapters are directed at both students and professors. The aim of each chapter is to cover recent advances and to provide up-to-date information, which is done well. Many of the citations at the end of each chapter are from recent work published in the literature. Related textbooks are also provided at the end of each chapter, to enable the reader to readily obtain additional information.

Overall this is an excellently written, comprehensive text that will be of interest to students and faculty alike. It can be skimmed to find areas of particular interest to the reader. The size of the book is reasonable to travel with in one's backpack or carry bag. Although the list price is $199, it is not hard to find it discounted to about $150 on the Internet.

## Suitability of the Text for Coursework and Research

The text is eminently readable, and would be quite suitable as an undergraduate course text. The course director might wish to supplement the text with another book with solved image processing problems. The text only includes specific chapters with expertise of the many authors. However, the entire field is rather well encompassed. Each chapter has a number of equations which the course director might want to cover in detail during class time. Many medical images, most in color, serve to illustrate each chapter well. This will therefore likely be an enjoyable book for students. At the graduate level, the book will provide basic information but should be supplemented with additional material that is more rigorous. Clinicians with a basic understanding of mathematical principles should find the book useful and up-to-date.

## Framework of the Book

The book includes an explanatory preface as well as a detailed table of contents which is subdivided, for each chapter, into sections and subsection. Thus it is possible to handily find specific aspects of the topic that are of interest to the reader. A list of acronyms and the book contributors is also provided. The editor writes the first chapter, entitled 'Fundamentals of Biomedical Image Processing'. Following this chapter, the book is also divided into Parts. Thus the major aspects of biomedical image processing are grouped together as chapters with similar focus. Lastly, there is a comprehensive index.

Chapter 1 is an overview of foundational material in biomedical image processing. It remarks on the steps used for processing, the terminology that is in current use, medical image formation, current imaging modalities, and basic concepts of image processing techniques. Included are quite a few diagrams and medical images which illustrate the concepts well. Typically there are a set of images or diagrams on every second or third page of the text.

Subsequent chapters follow the paradigm of summary, introduction, illustrated concepts (with some mathematics included), conclusions, and references. Again, quite a few of the medical images serving to illustrate the concepts are in full color. The images, flow diagrams, graphs, and tables that appear in the chapters are mostly original material not reprinted from scientific papers.

## Specific Content

In Part I of the text, image formation is covered in detail. The two particular aspects that are discussed thoroughly are hybrid imaging (fusion of PET and MRI) and cardiac 4D ultrasound imaging. The advantage of MRI is to enable detailed anatomic information to be acquired in the medical images. Whereas, PET is useful to obtain molecular and genetic information. By combining or fusing the two modalities, it is possible to determine the microscopic properties of tissues in the context of whole-organ location and function. Regarding cardiac 4D ultrasound imaging, until recently, conventional 2D ultrasound was the most commonly used modality, but the interpretation of these images can be difficult in the case of complex anatomic structures. Thus real-time volumetric imaging available using cardiac 4D ultrasound imaging is desirable, particularly for imaging moving structures such as the heart.

Part II covers topics in biomedical image enhancement, including morphological processing and image registration. Morphological considerations are important for filtering, segmentation, and pattern recognition. The types of morphological operations that are discussed include basic methods such as erosion, dilation, and opening and closing of image features. The problem of over-segmentation and how to prevent it is also discussed. Image registration is detailed in terms of transformation models that are applied so that morphed features, or images obtained with differing modalities, can be readily compared using similarity measures. Techniques for optimization of registration boundaries based upon the similarity measures are also discussed.

In Part III, feature extraction and selection are presented (texture, multiscale/multiorientation adjustments, and decision making). The texture analysis aspect is covered in terms of the specific operators and transformations that can be used for quantifying texture magnitude and properties. Multiscale and multiorientation adjustments are highly important to put image features in their proper context prior to features quantification, and are discussed in a separate chapter. Once whole-image quantification has been completed, the detection, extraction, and selection of features is possible. Decision-making to determine whether the extracted features are normal or abnormal in the context of clinical practice is then discussed.

Part IV covers segmentation algorithms, including clustering methods, methods to determine the degree of connectedness of different parts of an image, and the use of models as templates to discern different image areas. These topics include both parametric and nonparametric methods for clustering. The process of clustering is essential to group image components into contiguous regions. Region-based methods of segmentation are then covered, including methods of fuzzy connectedness of image regions. Specific algorithms that can be implemented computationally are provided in the text. Model-based segmentation is also enunciated, primarily by describing the use of deformable simplex meshes to determine segment boundaries.

Part V covers techniques to measure image properties, and to quantitatively classify images and image segments. This includes chapters that describe the automated detection of skin and breast cancer, and to analyze medical images as an aid to therapy. Diagnosis of malignant melanoma i.e. skin cancer, is an important medical image processing problem, with current levels of sensitivity and specificity being in the low-to-mid 90% range. The chapter covers topics for developing more efficacious computational tools. Clinical diagnosis of melanoma requires extensive training and experience; thus the need for a computational approach rather than simply using a qualitative clinical scoring for improved diagnosis. Breast cancer is a second major medical problem in which the efficacy of computationally-derived methods of detection and diagnosis is currently rather limited. Thus a chapter is devoted to new technology, including imaging with contrast-enhanced MRI for greater clarity of tissue structures. An overview of imaging biomarkers for drug development, along with the regulatory requirements, is discussed as a final chapter in this portion of the text.

In Part VI, methods to enhance images for visualization are discussed. Although quantitative methods are important for detection and estimation of salient features, satisfactory visualization is essential in order for the clinician to verify the findings of the quantifier. This part of the book includes chapters on segmentation of distinct anatomic structures that are captured over a sequence of medical images, and techniques for enhanced processing of MRI images, one of the most frequently acquired and useful medical image modalities. Volume rendering is presented, which is highly important to adequately visualize any segmented anatomic structures with complex topology, as for example, blood vessels. New exploratory methods such as virtual endoscopy are also discussed. Diffusion MRI, used for noninvasive investigation of neural architecture, is elucidated from the framework of volume segmentation, fiber clustering, and tissue analysis.

In Part VII is described the management and integration of images. This portion of the book includes chapters on Digital Imaging and Communications in Medicine (DICOM format), methods of computer-assisted detection, and fast retrieval of medical images based on content. The actual DICOM standard is described in more than 3,600 pages of technical documentation, which is condensed to a readable form in the text. A separate chapter is devoted to another major standard, the Picture Archiving and Communication System (PACS) - based method for computer-aided detection and diagnosis (CAD). A third topic important to management and integration of images is that of content-based image retrieval. This is essential for evaluating the large volume of data acquired from many patients over the course of a clinical workday and workweek. Several specific ongoing projects for this purpose are presented.

Finally in Part VIII, image evaluation and customization is discussed. This includes chapters on currently available toolkits and software, and problems in bringing new methods to the clinician. Once new algorithms for medical image processing are developed, ideally they need to be evaluated, compared and contrasted in terms of their overall relevance for clinical application. Tools for this purpose are also discussed in this portion of the text. Often, such algorithms are bundled in packages called toolkits, which are available commercially or as freeware, the topic of another chapter. Finally, the problem of bringing new imaging and image processing technology from the lab and computer to the hands of radiologists and other clinicians in a timely manner is discussed.

## Conclusions

Biomedical Image Processing, by Thomas M. Deserno, is an excellent book for both upper-level undergraduate coursework as well as for mid-level research scientists and clinicians interested in applying new methods to their medical imaging data. The text flows smoothly from one chapter to the next even though many authors were involved, and it is well-written. The information is mostly comprehensive and cohesive, and should provide helpful material to evaluate medical images and imaging modalities using the latest technology. The book can also serve as an informational device so that the latest research in the literature using new methods can be better understood. The size of the book is reasonable, and although the cost is somewhat high, the presence of plentiful medical color images will assist the reader in working with commonly acquired data in a clinical or laboratory setting.

## Competing interests

The author declares that he has no competing interests.

## Authors' contributions

EJC developed and wrote this review.

## References

[B1] Biomedical Image Processing Thomas M Deserno 2011 Springer-Verlag New York

